# Risk of psychiatric readmission in the homeless population: A 10-year follow-up study

**DOI:** 10.3389/fpsyg.2023.1128158

**Published:** 2023-02-15

**Authors:** Jesús Herrera-Imbroda, José Guzmán-Parra, Antonio Bordallo-Aragón, Berta Moreno-Küstner, Fermín Mayoral-Cleríes

**Affiliations:** ^1^Unidad de Gestión Clínica de Salud Mental, Instituto de Investigación Biomédica de Málaga (IBIMA), Hospital Regional Universitario de Málaga, Málaga, Spain; ^2^Departamento de Farmacología y Pediatría, Facultad de Medicina, Universidad de Málaga, Andalucía Tech, Málaga, Spain; ^3^Departamento de Personalidad, Evaluación y Tratamiento Psicológico, Facultad de Psicología, Universidad de Málaga, Andalucía Tech, Málaga, Spain

**Keywords:** homelessness, psychiatric readmission, health management, social psychiatry, psychopathology

## Abstract

Homelessness continues to be a major social and clinical problem. The homeless population has a higher burden of disease that includes psychiatric disorders. In addition, they have a lower use of ambulatory health services and a higher use of acute care. Few investigations analyze the use of services of this population group in the long term. We analyzed the risk of psychiatric readmission of homeless individuals through survival analysis. All admissions to a mental health hospitalization unit in the city of Malaga, Spain, from 1999 to 2005, have been analyzed. Three analyses were carried out: two intermediate analyses at 30 days and 1 year after starting follow-up; and one final analysis at 10 years. In all cases, the event was readmission to the hospitalization unit. The adjusted Hazard Ratio at 30 days, 1-year, and 10-year follow-ups were 1.387 (*p* = 0.027), 1.015 (*p* = 0.890), and 0.826 (*p* = 0.043), respectively. We have found an increased risk of readmission for the homeless population at 30 days and a decreased risk of readmission at 10 years. We hypothesize that this lower risk of long-term readmission may be due to the high mobility of the homeless population, its low degree of adherence to long-term mental health services, and its high mortality rate. We suggest that time-critical intervention programs in the short term could decrease the high rate of early readmission of the homeless population, and long-term interventions could link them with services and avoid its dispersion and abandonment.

## 1. Introduction

In the 20 years between the 1970s and 1990s, during the psychiatric reform, mental health hospital beds in Spain have been reduced by more than a quarter and long-term beds by a half (Aizpuru et al., 2008). A minority of hospitalized users make disproportionate use of inpatient mental health services (Vogel and Huguelet, 1997; Bowersox et al., 2012) which represents a wide part of the cost of mental health services. Homeless users have increased use of acute mental health services (Dickey et al., 1996) and emergency services (Currie et al., 2018). This excessive use of services represents an important economic burden and decreases the quality of care. However, it is known that they use fewer primary care services and community mental health services. Although there are several studies about readmissions in mental health users (Vogel and Huguelet, 1997) and homeless users (Laliberté et al., 2020), there are few studies that analyze the risk of readmissions of homeless users in the long term, and, to the best of our knowledge, there are no studies in Spain about mental health hospitalization readmission in the homeless population.

The homeless population in Spain has been traditionally a stigmatized population. In fact, in 1933 was approved the “Ley de vagos y maleantes” (Law of lazy and thugs) (Presidencia del Consejo de Ministros, 1933) for the control of beggars and ruffians with no known occupation. This law was not derogated until 1995 and was applied as an important repression instrument in the Franco regime. The homeless phenomenon in Spain has increased a 25% in the last decade, as shown by data from the INE [*Instituto Nacional de Estadística (Statistical National Institute*)], the main Spanish agency in charge of the statistical services of the State (INE, 2022). The rate is the same in Spanish and migrants. Mental health problems are frequent among them, with more than fifty percent with depressive symptoms (INE, 2022). It is estimated that more than 3 of 4 homeless have a mental disorder, being the most common substance use disorders and schizophrenia spectrum disorders (Gutwinski et al., 2021). Also, mental health problems increase vulnerability to this condition (Sullivan et al., 2000). However, the characteristics of the homeless population admitted to mental health hospitals are scarcely known and deserve to be more deeply studied (Kent et al., 1995).

The aim of this study was to analyze the characteristics of admissions in the homeless condition in a mental health hospitalization unit and analyze the risk of readmission at 10-year follow-up period and, as secondary outcome, at 1 year and 30 days from discharge. Studying the characteristics of mental health inpatients and the risk for readmission could be useful to design better specific interventions for this population.

## 2. Materials and methods

### 2.1. Setting

This study has been carried out in a mental health hospitalization unit located in Malaga (Andalusia, Spain), near the city center. The unit has 42 beds for a catchment area of approximately 500.000 inhabitants. The study population consisted of all hospital admissions that occurred during the study period. This unit is part of the mental health care system within the Andalusian Health Service, which provides universal health coverage to all people living in the autonomous community. This system prioritizes a community care model where hospital admission is considered a last resort when other measures have failed, or the outpatient approach is not possible. The mental health department of the hospital also comprises other units such as two community mental health centers, 1 day center, one medium- and long-stay ward (30 beds), one child and adolescent mental health unit, an intensive community treatment team, a care team for first episodes of psychosis, and an eating disorders unit. On the other hand, this unit maintains frequent coordination with a public foundation, FAISEM [*Fundación Andaluza para la Integración Social del Enfermo Mental (Andalusian Foundation for the Social Integration of the Mentally Ill*)]. It provides socio-health support to users with severe mental disorders, such as supervised houses, day centers, etc.

### 2.2. Ethics statements

The hospital Ethics Committee approved the study. Informed consent was not deemed necessary because the information used for the study was obtained retrospectively from computerized admissions records and anonymity was guaranteed.

### 2.3. Design and variables

The design of the study had two parts: a first recruitment period, which included all hospital admissions that took place from January 1, 1999, to December 31, 2005; and a second follow-up period, when we carried out an up-to-10-years follow-up of each included admission during the recruitment period. When readmission occurred during this follow-up, it ended. Although many users were able to have multiple hospitalizations, for our analysis we focused only in the time from each admission in the recruitment period to the next admission of the same user during the follow-up. The follow-up data of the patients rest exclusively on the hospital records. No active follow-up of the patients was done.

The sample was divided into two groups: admissions of homeless users; and admissions of resident users. A total of 5,538 hospital admissions were identified during the recruitment period. Of these, in 755 there was no information in the records consulted on whether they corresponded to homeless users. Therefore, a total of 4,783 valid cases were finally included in the analysis.

For the survival analysis, the primary outcome was the time between the initial admission and the first readmission during the 10 years follow-up period. The secondaries outcomes were the time between the initial admission and the first readmission during the 30 days and 1 year follow-up periods. Patient data with no readmission during the follow-up period are considered to be censored.

The independent variable of the study was homelessness condition recorded at the time of admission. Also, the following sociodemographic and clinical variables were recorded in each group: age, sex, length of stay, diagnosis, type of admission (urgent or scheduled), and legal status of admission (voluntary or involuntary). For the variable “diagnosis,” the different final diagnoses made by psychiatrists at discharge [ICD-10 (*International Clasification of Diseases 10th Edition*) diagnostic labels] were used. For the analysis, wide diagnosis categories were used. These categories were: “substance use disorders” (F10–F19), “bipolar disorders” (F31), “psychotic disorders” (F20–F29), “personality disorders (F60–F69)”, and “other disorders (F00–F09, F32–F39, F40–F49, F50–F59, F70–F79, F80–F9, F-90–F99)”.

### 2.4. Statistical analysis

A descriptive analysis of the variables was carried out in both groups. For the quantitative variables (age and length of stay) the mean and standard deviation were calculated and the differences between groups were analyzed using the Mann-Whitney *U* test as the distribution did not follow a normal distribution (Shapiro-Wilk test). For the qualitative variables (sex, diagnosis, type of admission, and legal status of admission) the frequency and percentage in each category were calculated and the differences between groups were analyzed using the Chi-Square test. Univariate survival analysis was carried out and a Kaplan-Meier curve was calculated. Three survival analyses were performed: two intermediate analyses at 30 days and 1 year; and one final analysis at 10 years. In all cases, the event was readmission to the hospitalization unit. Subsequently, a multivariate Cox regression analysis for each follow-up period (30 days, 1 year, and 10 years) was carried out. Survival analysis and Cox regression are very useful statistical tools used in life and health sciences when we want to measure time-to-event outcomes, as they offer more information than simply whether or not an event occurred (Benítez-Parejo et al., 2011; George et al., 2014).

We carried out an *a priori* analysis based on the literature consulted of those variables that could behave as potential confounders. Based on this analysis, in addition to the homelessness condition, variables sex, age (without statistically significant differences between the groups), and length of stay and diagnosis (with statistically significant differences between the groups) were included in the model. For the variable “diagnosis”, personality disorders were used as a reference category, since its effect on psychiatric readmission has already been established in previous studies (Vigod et al., 2015). Since we did three comparisons, we applied for the main outcome and for the secondary ones a Bonferroni correction and the significance threshold was set to α = 0.017 (α/3). SPSS version 25 (IBM Inc., Armonk, USA) was used to carried out the analyses.

## 3. Results

Of the total sample analyzed, 200 admissions (4.2%) corresponded to homeless users, and 4,583 admissions (95.8%) corresponded to resident. In the homeless group, the mean age was 39 years with a standard deviation of 11, and the mean length of stay was 10 days with a standard deviation of 14. In the resident group, the mean age was 39 years with a standard deviation of 13, and the mean length of stay was 12 days with a standard deviation of 14. For both groups, the most frequent categories for the variables sex, diagnosis, type of admission, and legal status of admission were, respectively, “male” (59.5% in homeless group; 66.1% in resident group), “psychotic disorders” (F20–F29) (27.1% in homeless group; 35.5% in resident group), “urgent admission” (92.5% in homeless group; 90.9% in resident group) and “involuntary admission” (84.9% in homeless group; 88.3% in resident group). Statistically significant differences between the groups were found for the variables length of stay and diagnosis. Detailed information regarding the sample is displayed in Table 1.

**TABLE 1:** Baseline variables.

		Homeless [*n* = 200 (4.2%)]	Resident [*n* = 4,583 (95.8%)]	*p* value
Age [Mean (SD)]		39 (11)	39 (13)	0.696^a^
Sex [n (%)]	Male	119 (59.5%)	3027 (66.1%)	0.054^b^
	Female	81 (40.5%)	1553 (33.9%)	
Length of stay [Mean (SD)]		10 (14)	12 (14)	**0.008^a^**
Diagnosis [n (%)]	Substance use disorders (F10–F19*)	39 (19.6%)	538 (11.8%)	**<0.001^b^**
	Bipolar disorders (F30,F31*)	36 (18.1%)	584 (12.8%)	
	Psychotic disorders (F20–F29*)	54 (27.1%)	1622 (35.5%)	
	Personality disorders (F60–F69*)	38 (19.1%)	413 (9.1%)	
	Other disorders (F00–F09, F32–F39, F40–F49, F50–F59, F70–F79, F80–F9, F-90–F99*)	32 (16.1%)	1406 (30.8%)	
Type of admission [n (%)]	Urgent admission	185 (92.5%)	4166 (90.9%)	0.440^b^
	Scheduled admission	15 (7.5%)	417 (9.1%)	
Legal status of admission [n (%)]	Voluntary admission	30 (15.1%)	531 (11.7%)	0.149^b^
	Involuntary admission	169 (84.9%)	4007 (88.3%)	

^a^*p* value from Mann-Whitney *U* test; ^b^*p* value from Chi-Square test; *Diagnostic labels from ICD-10 (International Classification of Diseases). Bold values correspond to significant results.

**FIGURE 1:**
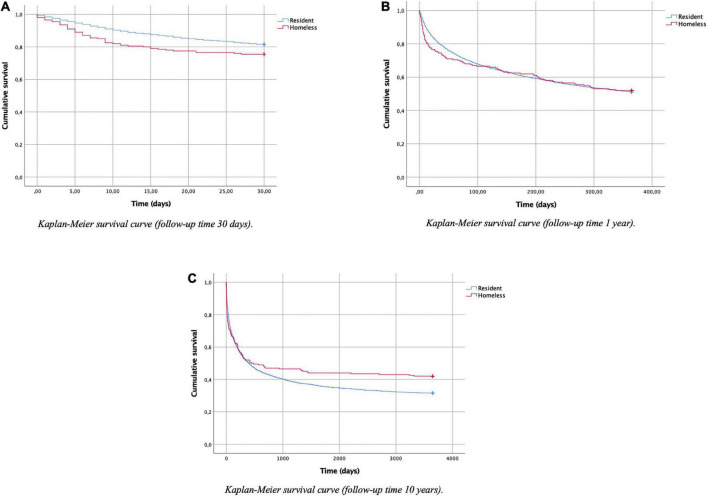
Kaplan-Meier survival analysis representation. **(A)** Kaplan-Meier survival curve (follow-up time 30 days). **(B)** Kaplan-Meier survival curve (follow-up time 1 year). **(C)** Kaplan-Meier survival curve (follow-up time 10 years).

For the univariate analysis, the results are shown in Table 2. Figure 1 represents the survival function using a Kaplan-Meier curve. For the multivariate analysis, a summary of these data can be found in Table 3 and detailed data can be found in Supplementary Tables 1–3. The diagnostic category “personality disorders” (F60–F69) was consistently associated with an increased risk of readmission, finding significant differences with the categories “substance use disorder” in all follow-up periods and with the category “psychotic disorders” at 30 days and 365 days of follow-up. Below we detail the most important findings for the main and secondary outcomes.

**TABLE 2:** Kaplan-Meier survival analysis.

	30 days			1 year			10 years		
	**N cases (%) of readmission**	Mean survival (CI 95%)*	uHR (95% CI)/ *p* value**	**N cases (%) of readmission**	Mean survival (CI 95%)*	uHR (95% CI)/ *p* value**	**N cases (%) of readmission**	Mean survival (CI 95%)*	uHR (95% CI)/ *p* value**
Homeless	49 (24.5%)	24.605 (23.216–25.994)	1.422 (1.066–1.897)/0.016	96 (48%)	228.440 (206.590–250.290)	1.022 (0.833–1.253)/0.836	116 (58%)	1695.145 (1456.491–1933.799)	0.835 (0.694–1.006)/0.057
Resident	846 (18.5%)	26.617 (26.387–26.848)		2236 (48.8%)	231.576 (227.168–235.984)		3134 (68.4%)	1425.221 (1378.782–1471.660)	
Overall	895 (18.7%)	26.533 (26.305–26.762)		2332 (48.8%)	231.445 (227.124–235.766)		3250 (67.9%)	1436.508 (1390.880–1482.136)	

*Mean survival in days **p value from Log-Rank (Mantel-Cox).

uHR, unadjusted Hazard Ratio; CI, confidence interval.

**TABLE 3:** Multivariate cox regression summary.

		30 days		1 year		10 years	
		**Exp(B) (95% CI)**	***p* value**	**Exp(B) (95% CI)**	***p* value**	**Exp(B) (95% CI)**	***p* value**
Age		0.985 (0.979–0.990)	0.000	0.991 (0.988–0.994)	0.000	0.989 (0.987–0.992)	0.000
Sex	Male	1.087 (0.942–1.255)	0.253	1.116 (1.020–1.222)	0.017	1.118 (1.036–1.206)	0.004
	Female (reference)	–	–	–	–	–	–
Length of stay		1.004 (1–1.008)	0.070	1.004 (1.001–1.006)	0.002	1.005 (1.003–1.007)	0.000
Diagnosis	Substance use disorders	0.608 (0.466–0.794)	0.000	0.744 (0.626–0.885)	0.001	0.794 (0.683–0.923)	0.003
	Bipolar disorders	0.826 (0.638–1.070)	0.148	0.900 (0.758–1.069)	0.230	1.073 (0.926–1.243)	0.347
	Psychotic disorders	0.518 (0.415–0.647)	0.000	0.808 (0.699–0.933)	0.004	0.940 (0.829–1.066)	0.336
	Personality disorders (reference)	–	–	–	–	–	–
	Other disorders	0.861 (0.696–1.065)	0.168	0.834 (0.719–0.967)	0.016	0.828 (0.727–0.943)	0.004
Homeless	Yes	1.387 (1.308–1.853)	0.027	1.015 (0.827–1.245)	0.890	0.826 (0.686–0.994)	0.043
	No (reference)	–	–	–	–	–	–

Exp (B) = adjusted Hazard Ratio. CI, confidence interval.

### 3.1. 10 years follow-up (main outcome)

The frequency (and percentage) of readmission was 116 cases (58%) for the homeless group and 3,134 cases (68.4%) for the resident group.

In the univariate analysis for the 10-year follow-up period, an unadjusted Hazard Ratio (uHR) of 0.835 [95% CI = (0.694–1.006)] was calculated for the homeless group. The mean survival time was 1695.145 days [95% CI = (1456.491–1933.799)] for the homeless group; and 1425.221 days [95% CI = (1378.782–1471.660)] for the resident group. These differences were not statistically significant on the test of equality of survival distributions (Log Rank *p* value = 0.057).

In the multivariate analysis for the 10-year follow-up period, an adjusted Hazard Ratio (aHR) of 0.826 [95% CI = (0.686–0.994)] was calculated for the homeless factor. In this model, statistical significance was only nominally achieved, with a *p*-value of 0.043. As for other categorical variables present in the Cox regression, differences in sex and some diagnostic categories were statistically significant.

### 3.2. 30 days follow-up (secondary outcome)

The frequency (and percentage) of readmission was 49 cases (24.5%) for the homeless group and 846 cases (18.5%) for the resident group. In the univariate analysis for the 30-days follow-up period, an unadjusted Hazard Ratio (uHR) of 1.422 [95% CI = (1.066–1.897)] was calculated for the homeless group. The mean survival time was 24.605 days [95% CI = (23.216–25.994)] for the homeless group; and 26.617 days [95% CI = (26.387–26.848)] for the resident group. These differences were statistically significant on the test of equality of survival distributions (Log Rank *p* value = 0.016).

In the multivariate analysis for the 30-days follow-up period, an adjusted Hazard Ratio (aHR) of 1.387 [95% CI = (1.038–1.853)] was calculated for the homeless group. In this model, marginal statistical significance was achieved, with a *p*-value of 0.027. As for other categorical variables present in the Cox regression, differences in some diagnostic categories were statistically significant.

### 3.3. 1-year follow-up (secondary outcome)

The frequency (and percentage) of readmission was 96 cases (48%) for the homeless group and 2,236 cases (48.8%) for the resident group.

In the univariate analysis for the 1-year follow-up period, an unadjusted Hazard Ratio (uHR) of 1.022 [95% CI = (0.833–1.253)] was calculated for the homeless group. The mean survival time was 228.440 days [95% CI = (206.590–250.290)] for the homeless group; and 231.576 days [95% CI = (227.168–235.984)] for the resident group. These differences were not statistically significant on the test of equality of survival distributions (Log Rank *p* value = 0.836).

In the multivariate analysis for the 1-year follow-up period, an adjusted Hazard Ratio (aHR) of 1.015 [95% CI = (0.827–1.245)] was calculated for the homeless factor. In this model, statistical significance was not achieved, with a *p*-value of 0.890. As for other categorical variables present in the Cox regression, differences in sex and some diagnostic categories were statistically significant.

## 4. Discussion

It is well-established that, in the short term, the homeless population is more likely to be readmitted to a psychiatric inpatient unit than resident, especially within 30 days from discharge (Laliberté et al., 2020; Mascayano et al., 2022). In our sample, we found a similar risk in comparison with those described in previous studies (although after the Bonferroni correction, the differences in the multivariate analysis were only marginally significant). This is important, as early readmission is a negative outcome from a clinical and public health perspective, and many efforts of clinicians and researchers have been put into developing interventions that reduce early readmission (Vigod et al., 2013, 2015). In a recent review, Owusu et al. (2022) list some of these interventions: residential treatment services, adequate and sufficient hospital care, establishing an adequate discharge plan (discharge services, follow-up calls, short-term case management, bridge visits, and psychoeducation), focusing on staff training and coordination of care and transition efforts, provide psychological support (including proper addressing of patients’ perceived needs) and ensure medication adherence (Owusu et al., 2022).

Homelessness is a condition that confers on those who suffer it a significant personal vulnerability, having been described as part of a “fourth world” (Raps and Kemelman, 1994), or third world within the first world. Homeless users have a high prevalence of both physical and mental illnesses (Fazel et al., 2008, 2014), as well as poor access to primary care services (Khandor et al., 2011) and ambulatory mental health services (Folsom et al., 2005). All of this makes them more likely to use acute care services (Chambers et al., 2013; Saab et al., 2016). This, added to the fact that shelters are not appropriate places to recover from an episode of mental illness requiring hospitalization (Forchuk et al., 2006), generates a “perfect storm” that would explain the high rates of early psychiatric readmission found in this population. This is also supported by the fact that the lack of social support at discharge and the absence of availability of housing solutions are predictors of psychiatric readmission (Scanlan et al., 2017; de Jong et al., 2021). So, many of these homeless users may experience the “revolving door” phenomenon, which indicates repeated hospitalizations of the same patients, and which has become a public health problem (Doran et al., 2013; di Giovanni et al., 2020). Some authors have described homeless patients with mental illnesses as “super-difficult” patients, object of Marontology, an unborn medical specialty recently proposed (Gama Marques, 2021).

In our sample, we found a higher mean length of stay in the resident group than in the homeless group (12 vs. 10 days respectively). This is an unexpected finding since, in general, the literature states that homeless users on medical and surgical services remain hospitalized longer than housed users, resulting in substantial excess costs (Hwang et al., 2011). For us, a possible explanation is that in our city we have a municipal shelter with which we work in a coordinated manner and that generally accepts homeless patients when they leave the hospital, in a relatively fast time. Therefore, the problem would not be so much whether our homeless population has a place to live at hospital discharge, but whether or not this site is suitable for their health needs.

On the other hand, both in the homeless group and in the resident group, the most frequent diagnostic category was “psychotic disorders” (27.1 and 35.5% respectively). This is an expected fact since we are talking about a population that has been admitted to a psychiatric hospitalization unit. However, it is noteworthy that, while in the resident group the second most frequent diagnostic category is “other disorders” (30.8%), a large group that includes mental disorders with a better prognosis such as depressive or anxiety disorders, in the homeless group this place is occupied by the “substance use disorders” (19.6%) followed closely by “personality disorders” (19.1%). Considering that these disorders constitute common debilitating conditions which increase the risk of all-cause mortality (Smith and Cottler, 2020), our finding would support what has been referred to in previous studies on the high burden of disease in the homeless collective.

However, despite these findings, there are not many studies that analyze the psychiatric readmission risk in the homeless population in the long term. Our study analyzes the time to readmission in all episodes of hospitalization of homeless and resident psychiatric users, with a follow-up period of up to 10 years. And it does so from the perspective of a single inpatient unit. In this sense, we have found that, as the follow-up period increases, the greater risk of readmission of the homeless population decreases. Thus, this is equated in the analysis of survival to 1 year with the resident population and even could decrease at 10 years. Although we have to be prudent in the interpretation of these results (since some these differences were only nominally or marginally significant after the Bonferroni correction and the multivariate analysis), we think that it shows a tendency which can have several explanations.

So, for this phenomenon we hypothesize three possible causes: the mobility of the homeless population, their disengagement from mental health services, and the high mortality of this group.

The reality of homeless mobility is a controversial issue, with an older body of evidence suggesting high residential transience in this population (Bachrach, 1987; Pollio, 1997; Duchon et al., 1999), most questioned today (Parker and Dykema, 2013). It is possible that the differences found are due to a heterogenous definition of the concept of transience, the geographical area of study, and an improvement over time in the social resources available to the homeless population. In our case, Malaga is a city well-connected with many other nearby places and quasi-border with other countries such as Morocco, being a place of habitual passage of a significant proportion of the migrant population, many of them with very limited economic resources. Therefore, it is quite likely that the homeless population that habitually or temporarily resides in our city has a high level of instability residence. In any case, recent studies show that adults with residential transience had greater odds of mental illness than those without transience (Glasheen et al., 2019).

On the other hand, after psychiatric discharge, homeless users are less likely to have adequate medical follow-up (Burra et al., 2012), and they have difficulties in long-term engaging with services and having an adequate level of commitment to treatment (Dixon et al., 2016). Thus, while early psychiatric readmission can be a reliable indicator of unsatisfied needs at discharge; in the long term, the fact that a subject with a severe mental illness disappears from the medical records of a hospital could be indicating a complete abandonment of the use of mental health services, and an inability of these to detect this population at risk and care for it adequately.

Finally, we need to consider the high mortality rate of homeless users compared to the general population (Aldridge et al., 2018), which may have to do with various factors, such as increased disease burden or aging (Fazel et al., 2014). Since we have used only clinical records of admissions and discharges, in one psychiatric hospitalization unit, it is plausible that the differences found in the long term are due to higher mortality and mobility in this population.

## 5. Limitations

In this work we have tried to shed some light on the complex problems that homelessness represents for acute mental health services, and on its complex relationship with psychiatric admissions and readmissions. Although we consider that some interesting conclusions can be drawn from our results, as we relate in the following section, we cannot abstract from the limitations of our study. First, some results, once Bonferroni correction for multiple comparisons has been performed, reach only nominal or marginal statistical significance. This may be because we do not have a very large sample size. Secondly, although we have calculated for both univariate and multivariate analysis the size of the effect through the Hazard Ratio, the clinical relevance of the results could be discussed. Also, the fact of having focused only on the readmissions that have occurred during a specific period in a single hospital, means that we do not have all the information we would like about the future of these users in terms of mortality, geographical mobility, or admissions in different hospitalization units, having to make hypotheses about these aspects. Furthermore, no active follow-up of the patients was done. Finally, we do not have exact information on the percentage of admissions in the homeless group that actually correspond to the migrant population, which would help us to contrast the hypothesis about their high mobility and disengagement with local mental health services. We don’t know either the percentage of anonymous patients, which would allow us to compare with recent studies about the John Doe syndrome (Gama Marques and Bento, 2020).

## 6. Conclusion

Homelessness remains a major social problem with significant clinical and public health implications. Our study shows in line with other previous studies that the risk of early readmission in the homeless population is higher than in the resident population, which may be due to the greater psychic and somatic morbidity existing in this group at risk and the inexistence of appropriate resources for recovery to discharge.

However, when we analyze the behavior of the homeless population in the long term, these differences begin to blur, and the risk of long-term readmission to the same hospital tends to be lower than in the resident population, even when adjusted for potential confounding variables in the multivariate analysis. A possible limitation of our study is that we are only looking at what happened in only one hospitalization unit. However, interesting conclusions can also be drawn from this. We hypothesize, which should be confirmed in subsequent studies, that these differences could be justified by the high mobility of the homeless population of our city, its low degree of linkage with long-term mental health services, and the high mortality rate of this population group.

Finally, we believe that these should have a direct impact on health management and planning. On the one hand, to develop time-critical intervention programs in the short term to avoid the high rate of early readmission of the homeless population. On the other hand, to be able to link the homeless population in the long term and avoid its dispersion and abandonment as well as the generation of unsatisfied health needs.

## Data availability statement

The data analyzed in this study is subject to the following licenses/restrictions: The dataset could be available under reasonable request. Requests to access these datasets should be directed to JG-P, jose.guzman.parra.sspa@juntadeandalucia.es.

## Ethics statement

The studies involving human participants were reviewed and approved by Comité de Ética de la Investigación Provincial de Málaga. Written informed consent for participation was not required for this study in accordance with the national legislation and the institutional requirements.

## Author contributions

JH-I and JG-P participated in the conception, design, data analysis, and wrote the manuscript. AB-A and BM-K contributed to the conception and design of the study and its review. FM-C the senior author was active in the conception, design, and writing and edition of the manuscript. All authors approved the final version of the manuscript.
