# Supplementary material for: Risk of psychiatric readmission in the homeless population: A 10-year follow-up study
Source: Front Psychol. 2023 Feb 15;14:1128158. doi: 10.3389/fpsyg.2023.1128158 (PMC9975390; doi:10.3389/fpsyg.2023.1128158)
Supplement: Supplementary file 1 [file Table_1.DOCX]

Supplementary Material

# Supplementary Tables

| **Table S1** |  |  |  |  |  |  |  |  |  |
| --- | --- | --- | --- | --- | --- | --- | --- | --- | --- |
| *Multivariate Cox regression (follow-up time 30 days).* | | | | | | | | | |
|  |  |  |  |  |  |  |  | **95% CI for Exp (B)** | |
|  |  | **B** | **SE** | **Wald** | **df** | **p value** | **Exp (B)** | **Lower** | **Higher** |
| **Age** |  | -0,015 | 0,003 | 28,009 | 1 | 0,000 | 0,985 | 0,979 | 0,990 |
| **Sex (Male/Female)** |  | 0,084 | 0,073 | 1,309 | 1 | 0,253 | 1,087 | 0,942 | 1,255 |
| **Diagnosis** | **Personality disorders (reference)** |  |  | 51,097 | 4 | 0,000 |  |  |  |
|  | **Substance use disorders** | -0,497 | 0,136 | 13,319 | 1 | 0,000 | 0,608 | 0,466 | 0,794 |
|  | **Psychotic disorders** | -0,657 | 0,113 | 33,618 | 1 | 0,000 | 0,518 | 0,415 | 0,647 |
|  | **Bipolar disorders** | -0,191 | 0,132 | 2,096 | 1 | 0,148 | 0,826 | 0,638 | 1,070 |
|  | **Other disorders** | -0,150 | 0,109 | 1,900 | 1 | 0,168 | 0,861 | 0,696 | 1,065 |
| **Length of stay** |  | 0,004 | 0,002 | 3,294 | 1 | 0,070 | 1,004 | 1,000 | 1,008 |
| **Homeless (Yes/No)** |  | 0,327 | 0,148 | 4,900 | 1 | 0,027 | 1,387 | 1,038 | 1,853 |

*B=Coefficient;; CI=Confidence interval; df=Degrees of freedom; SE=Standard error; W=Wald test.*

*Exp (B) = Hazard Ratio.*

| **Table S2** |  |  |  |  |  |  |  |  |  |
| --- | --- | --- | --- | --- | --- | --- | --- | --- | --- |
| *Multivariate Cox regression (follow-up time 1 year).* | | | | | | | | | |
|  |  |  |  |  |  |  |  | **95% CI for Exp (B)** | |
|  |  | **B** | **SE** | **Wald** | **df** | **p value** | **Exp (B)** | **Lower** | **Higher** |
| **Age** |  | -0,009 | 0,002 | 27,097 | 1 | 0,000 | 0,991 | 0,988 | 0,994 |
| **Sex (Male/Female)** |  | 0,110 | 0,046 | 5,728 | 1 | 0,017 | 1,116 | 1,020 | 1,222 |
| **Diagnosis** | **Personality disorders (reference)** |  |  | 13,962 | 4 | 0,007 |  |  |  |
|  | **Substance use disorders** | -0,295 | 0,089 | 11,125 | 1 | 0,001 | 0,744 | 0,626 | 0,885 |
|  | **Psychotic disorders** | -0,214 | 0,074 | 8,439 | 1 | 0,004 | 0,808 | 0,699 | 0,933 |
|  | **Bipolar disorders** | -0,105 | 0,088 | 1,441 | 1 | 0,230 | 0,900 | 0,758 | 1,069 |
|  | **Other disorders** | -0,182 | 0,076 | 5,784 | 1 | 0,016 | 0,834 | 0,719 | 0,967 |
| **Length of stay** |  | 0,004 | 0,001 | 9,667 | 1 | 0,002 | 1,004 | 1,001 | 1,006 |
| **Homeless (Yes/No)** |  | 0,014 | 0,105 | 0,019 | 1 | 0,890 | 1,015 | 0,827 | 1,245 |

*B=Coefficient;; CI=Confidence interval; df=Degrees of freedom; SE=Standard error; W=Wald test.*

*Exp (B) = Hazard Ratio.*

| **Table S3** |  |  |  |  |  |  |  |  |  |
| --- | --- | --- | --- | --- | --- | --- | --- | --- | --- |
| *Multivariate Cox regression (follow-up time = 10 years).* | | | | | | | | | |
|  |  |  |  |  |  |  |  | **95% CI for Exp (B)** | |
|  |  | **B** | **SE** | **Wald** | **df** | **p value** | **Exp (B)** | **Lower** | **Higher** |
| **Age** |  | -0,011 | 0,001 | 55,983 | 1 | 0,000 | 0,989 | 0,987 | 0,992 |
| **Sex (Male/Female)** |  | 0,111 | 0,039 | 8,226 | 1 | 0,004 | 1,118 | 1,036 | 1,206 |
| **Diagnosis** | **Personality disorders (reference)** |  |  | 30,617 | 4 | 0,000 |  |  |  |
|  | **Substance use disorders** | -0,231 | 0,077 | 9,000 | 1 | 0,003 | 0,794 | 0,683 | 0,923 |
|  | **Psychotic disorders** | -0,062 | 0,064 | 0,926 | 1 | 0,336 | 0,940 | 0,829 | 1,066 |
|  | **Bipolar disorders** | 0,070 | 0,075 | 0,885 | 1 | 0,347 | 1,073 | 0,926 | 1,243 |
|  | **Other disorders** | -0,189 | 0,066 | 8,082 | 1 | 0,004 | 0,828 | 0,727 | 0,943 |
| **Length of stay** |  | 0,005 | 0,001 | 30,196 | 1 | 0,000 | 1,005 | 1,003 | 1,007 |
| **Homeless (Yes/No)** |  | -0,192 | 0,095 | 4,093 | 1 | 0,043 | 0,826 | 0,686 | 0,994 |

*B=Coefficient;; CI=Confidence interval; df=Degrees of freedom; SE=Standard error; W=Wald test.*

*Exp (B) = Hazard Ratio.*
